# Prediction of the survival and functional ability of severe stroke patients after ICU therapeutic intervention

**DOI:** 10.1186/1471-2377-8-24

**Published:** 2008-06-26

**Authors:** Moussa Riachy, Frida Sfeir, Ghassan Sleilaty, Samer Hage-Chahine, Georges Dabar, Taha Bazerbachi, Zeina Aoun-Bacha, Georges Khayat, Salam Koussa

**Affiliations:** 1 Department of Pulmonary and Critical Care Medicine, Hotel Dieu de France, Beirut, Lebanon; 2 Department of Cardiothoracic Surgery, Hotel Dieu de France, Beirut, Lebanon; 3 Department of Neurology, Hotel Dieu de France, Beirut, Lebanon

## Abstract

**Background:**

This study evaluated the benefits and impact of ICU therapeutic interventions on the survival and functional ability of severe cerebrovascular accident (CVA) patients.

**Methods:**

Sixty-two ICU patients suffering from severe ischemic/haemorrhagic stroke were evaluated for CVA severity using APACHE II and the Glasgow coma scale (GCS). Survival was determined using Kaplan-Meier survival tables and survival prediction factors were determined by Cox multivariate analysis. Functional ability was assessed using the stroke impact scale (SIS-16) and Karnofsky score. Risk factors, life support techniques and neurosurgical interventions were recorded. One year post-CVA dependency was investigated using multivariate analysis based on linear regression.

**Results:**

The study cohort constituted 6% of all CVA (37.8% haemorrhagic/62.2% ischemic) admissions. Patient mean(SD) age was 65.8(12.3) years with a 1:1 male: female ratio. During the study period 16 patients had died within the ICU and seven in the year following hospital release.

The mean(SD) APACHE II score at hospital admission was 14.9(6.0) and ICU mean duration of stay was 11.2(15.4) days. Mechanical ventilation was required in 37.1% of cases. Risk ratios were; GCS at admission 0.8(0.14), (p = 0.024), APACHE II 1.11(0.11), (p = 0.05) and duration of mechanical ventilation 1.07(0.07), (p = 0.046). Linear coefficients were: type of CVA – haemorrhagic versus ischemic: -18.95(4.58) (p = 0.007), GCS at hospital admission: -6.83(1.08), (p = 0.001), and duration of hospital stay -0.38(0.14), (p = 0.40).

**Conclusion:**

To ensure a better prognosis CVA patients require ICU therapeutic interventions. However, as we have shown, where tests can determine the worst affected patients with a poor vital and functional outcome should treatment be withheld?

## Background

Cerebrovascular accidents (CVA) are currently the second most common cause of mortality in the world, and remain the most common cause of long-term disability in adults [1-4]. New therapeutic methods are being advanced to limit ischemic neuronal damage, although their benefits are debatable [5-8]. The setting up of intensive care units (ICU) for the treatment of acute CVAs has caused controversy for many years [9-15], although new evidence has established an increased patient survival rate and quality of life in ICU treated patients compared to those treated in medical departments [3,4,9-14]. CVA patient admission into ICUs has now become routine, but can not ensure better survival or provide less physical or mental dependence for every patient. After suffering a catastrophic neurological event the benefits of intensive care remain controversial, and may only serve to prolong the period before the patient's inevitable death [16].

The study aim was to predict the benefit and impact of various ICU therapeutic interventions on the survival and functional abilities of CVA patients who had suffered severe ischemic or haemorrhagic stroke.

## Methods

This study was conducted at the Hôtel-Dieu de France university hospital in Beirut (Lebanon), linked to Saint-Joseph University, which has 8 intensive care beds for emergency and medical services.

A study was made of 62 ICU patients suffering from severe ischemic or haemorrhagic CVA entering the ICU (via the emergency or neurology departments, or other hospital departments) over a retrospective period of 30 months (July 2000 to January 2003) and 12 months prospectively (January 2003 to January 2004). Exclusion criteria included patients who had suffered from sub-arachnoidal haemorrhage, extra-dural haematoma or transient ischemic attack.

CVA severity was measured using an acute physiology, age and chronic health evaluation (APACHE II: range 0 to 71) [17] and a Glasgow coma scale (GCS: range 3 to 15) neurological assessment [18,19] at admission, 48 hours after admission and at ICU discharge. Patient survival was determined using Kaplan-Meier survival tables. The patients' state of dependence and functional abilities were measured using stroke impact scale-16 (SIS-16) [20,21], which provides a measurement of a patient's capacity to perform daily activities. Additionally, their general health status was assessed using a Karnofsky Score (range 0 to 100) [22,23] before ICU admission, at hospital discharge, and at 3 months and 1 year post-CVA.

Continuous variables were expressed as means with their standard deviation (SD), whereas categorical variables were expressed as actual numbers and percentages. For quantitative variables, a student *t*-test was used to compare the means of two categories. For more than two categories, an analysis of variance was used, and if there were statistically significant differences a post-hoc test was performed. Categorical variables were analyzed using a Chi-square test. A repeated-measures ANOVA was made to study the evolution of quantitative variables over time, and a Spearman correlation was used to study the relationship between neurological and general health severity scores.

Determination of independent variables predicting a CVA patient's physical dependency at discharge and after one year was made using a univariate analysis, supplemented by a multivariate linear regression.

Survival analysis tables were used to assess CVA patient survival. Univariate qualitative variables were compared using the log rank method. A multivariate analysis of survival predictive factors was made using the Cox proportional hazards technique. All predictive factors with a univariate p < 0.1 were entered in the multivariate model. To adjust for testing multiple hypotheses we performed the Holm stepdown Sidak procedure for multiple comparisons (P' sds). All other tests were considered statistically significant for a p-value < 0.05.

The statistical software used in this study was SPSS v13 (SPSS Inc, Chicago, Illinois).

## Results

Of the 62 patients studied, 47 (75.8%) came from the emergency department, 5 (8.1%) from the neurology department, 5 (8.1%) came from other medical departments within the hospital and 5 (8.1%) were transferred from another hospital. The male: female ratio was 1:1 with a mean (SD) age of 65.8 (12.3) years.

Thirty three patients (53.2%) suffered from embolic CVA, 17 (27.4%) from haemorrhagic CVA and 12 (19.4%) from thrombotic CVA. Half the CVA cases were in the carotid artery territory, 33.9% were in the vertebro-basilar artery territory and the remaining cases were in the cortical and subcortical areas. Risk factors (infection and cardiac problems) were identical for both ischemic and haemorrhagic stroke, except for atrial fibrillation which was found in 12 patients with ischemic stroke (p < 0.001).

At admission the mean APACHE II values, Karnofsky score and the SIS-16/80 were 14.90 (6.0), 91.50 and 74.93 respectively, which indicated a good initial functional state. The majority of patients were completely independent with an average Karnofsky score higher than 90 for both ischemic and haemorrhagic CVA. The GCS/15 at admission had a mean of 9.9.

A univariate comparison between survivors and non-survivors with respect of all risk factors studied was made and is presented in Table 1. Continuous normal data are represented as mean(SD), continuous non-normal data and ordinal data are represented as median (1st–3rd quartile) and categorical data are represented as frequencies.

**Table 1:** Univariate comparison between survivors and non-survivors with respect of all risk factors studied

	**Survivors (n = 46)**	**Non survivors (n = 16)**	**p-value**	**P' sds**
**Age (years)**	63.9 ± 11.7	71.1 ± 13.0	0.043	0.681
**Female gender**	50.0%	50.0%	0.999	1.000
**Hypertension**	71.7%	56.3%	0.254	0.995
**Diabetes mellitus**	23.9%	37.5%	0.294	0.996
**Dyslipidemia**	28.3%	12.5%	0.205	0.990
**COPD**	2.2%	0.0%	0.999	1.000
**Myocardial infarction**	10.9%	18.8%	0.418	0.999
**CABG**	4.3%	6.3%	0.999	1.000
**Cardiac failure**	6.5%	25.0%	0.044	0.675
**Atrial fibrillation**	17.4%	25.0%	0.507	0.999
**Renal failure**	19.6%	12.5%	0.524	0.999
**Cancer**	2.2%	6.3%	0.453	0.999
**Smoking**	30.4%	12.5%	0.158	0.977
**Hemorrhagic stroke**	26.1%	31.3%	0.690	0.999
**Previous stroke**	26.1%	25%	0.932	1,000
**Thrombolysis**	21.7%	0%	0.042	0.712
**Karnofsky score on admission**	100 (100–100)	80 (80–100)	0.029	0.621
**SIS 16/80 score on admission**	80 (80–80)	80 (70–80)	0.290	0.997
**Apache score on admission**	13 (10–16)	20 (18–23)	< 0.001	0.037
**GCS score on admission**	11 (9–13)	6 (4–10)	< 0.001	0.036
**Apache score after 48 hours**	12 (8–18)	19 (17–24)	0.003	0.097
**GCS score after 48 hours**	12 (9–14)	8 (4–11)	0.001	0.035
**Sedative treatment**	4.3%	25.0%	0.034	0.669
**Anti-hypertensive treatment**	65.2%	75.0%	0.471	0.999
**Anti-coagulant treatment**	78.3%	62.5%	0.215	0.990
**Anti-epileptic drugs**	15.2%	37.5%	0.059	0.768
**Diuretics**	17.4%	438%	0.034	0.658
**Corticosteroids**	13.0%	0.0%	0.325	0.997
**Gastric tube**	43.5%	87.5%	0.002	0.068
**Surgery**	4.3%	18.8%	0.103	0.918
**Infection**	43.5%	62.5%	0.190	0.988
**Cardiac complications**	19.6%	25.0%	0.646	0.999
**Tracheostomy**	10.9%	6.3%	0.999	0.999
**Respiratory physiotherapy**	15.2%	6.3%	0.357	0.998
**Mechanical ventilation duration**	9 (8–13)	5 (2–16)	0.554	0.998
**ICU length of stay**	4 (2–12)	9 (4–21)	0.042	0.699
**Apache score on ICU discharge**	10 (5–14)	16 (15–20)	0.040	0.706
**GCS score on ICU discharge**	13 (11–15)	11 (8–11)	0.042	0.686

COPD: Chronic obstructive pulmonary disease; CABG: Coronary artery bypass grafting. GCS: Glasgow coma scale. ICU: Intensive care unit. Continuous normal data are represented as mean ± standard deviation. Continuous non normal data and ordinal data are represented as median (1^st ^quartile – 3^rd ^Quartile); Categorical data are represented as frequencies (Percentage). P'sds is the adjusted P value derived from the Holm-Sidak step-down procedure to correct for multiple comparisons.

The ICU interventions and their significance in treating ischemic and haemorrhagic CVA are presented in Table 2. Additionally, it was found that; 10 (16.1%) patients in the ischemic group received thrombolysis, 34 (54.8%) had received a naso-gastric tube to avoid difficulties in swallowing, 23 (37%) underwent mechanical ventilation and five patients (8.1%) underwent surgical intervention.

**Table 2:** The number and percentage of ICU interventions for ischemic and haemorrhagic CVA and their significance (p-value)

**Intervention**	**Ischemic**		**Haemorrhagic**		**p-value**	**P' sds**
			
	N	%	N	%		
**Sedation**	2	4.45	4	23.53	0.043	0.296
**Anti-hypertensives**	28	62.22	14	82.35	0.223	0.717
**Anti-coagulants**	42	93.33	4	23.53	0.001	0.011
**Anti-epileptics**	4	8.89	9	52.94	0.001	0.010
**Osmotic diuretics**	8	17.78	7	41.18	0.094	0.499
**Steroids**	4	8.89	2	11.76	0.662	0.987
**Nasogastric tube**	22	48.89	12	70.59	0.159	0.646
**Surgery**	1	2.22	4	23.52	0.018	0.151
**Mechanical ventilation**	16	35.56	7	41.18	0.771	0.988
**Infections**	21	46.67	9	52.94	0.778	0.951
**Cardiac complications**	10	22.22	3	17.64	1.000	1.000

P' sds is the adjusted p value derived from the Holm step-down Sidak procedure for multiple comparisons.

**Figure 1:**
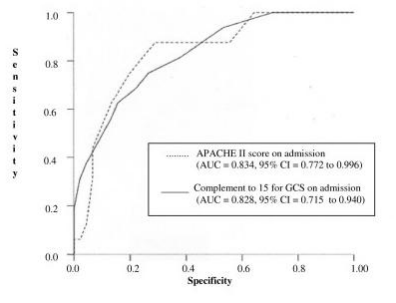
Receiver operating characteristic curves (ROC) for APACHE II and GCS at admission.

A receiver operating characteristic curves (ROC) was plotted for APACHE II and GCS; the area under the ROC curve was 0.834 (95% CI: 0.722 to 0.946) (p < 10^-3^) for APACHE II and 0.828 (95% CI: 0.715 to 0.940) (p < 10^-3^) for GCS. When the APACHE II and GCS curves are compared, the area under the ROC curve of APACHE II showed no significant differences in p-values. (Fig. 1).

### Disability assessments

**Figure 2:**
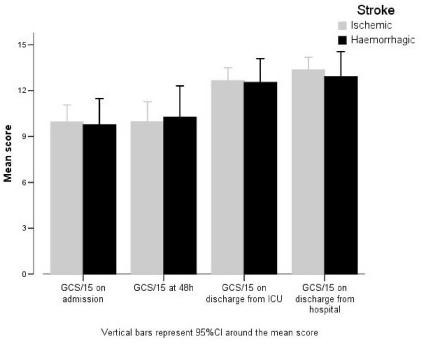
The Glasgow Coma Scale (GCS/15)

The GCS/15 was used to assess the depth and duration of a patient's coma and impaired consciousness (Figure 2). Results showed (*) a slow increase after admission and then a major increase between 48 hours post-CVA and ICU departure. After discharge from hospital there was a continuous but less marked improvement in the GCS.

**Figure 3:**
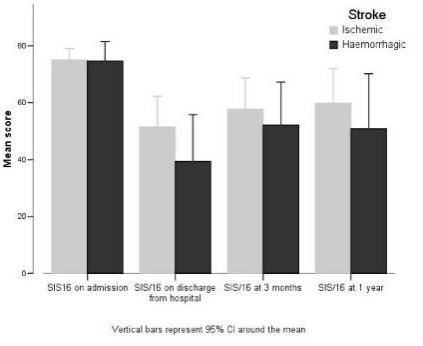
The Stroke Impact Scale 16 (SIS-16)

The SIS-16 was used to provide an indication of a patient's state of dependency (Figure 3). Results showed (*) a strong improvement after hospital departure and in the three months following, although this improvement was less pronounced later. The Karnofsky score, which was used to give an indication of the patient's functional status, showed a similar evolution to the SIS-16 for both types of CVA.

Univariate analysis indicated that the patient's functional state (SIS-16) at discharge from hospital was influenced by age, GCS at hospital admission, type of CVA and duration of hospital stay, whereas other variables had little influence. Multivariate analysis incorporating linear regression was used to determine the definitive significant factors (Table 3).

**Table 3:** Factors that influence a patient's functional state and dependency at discharge from hospital; multivariate analysis

	**Linear coefficient**	**Standard Error**	**p-value**
**(constant)**	14.991	12.425	0.273
**GCS at admission**	6.833	1.077	0.001
**Type of CVA Haemorrhagic vs Ischemic**	-18.954	4.579	0.007
**Duration of hospital stay (day)**	-0.378	0.144	0.040

One year after discharge, disability was essentially related to the SIS-16 at hospital discharge (Odds ratio (OR): 0.734(0.082) for ischemic and 0.374(0.024) for haemorrhagic stroke). Complementary factors in the ischemic group were: patient age (OR: -0.534(0.190)), mechanical ventilation (OR: -10.921(5.238)) and diabetes mellitus (OR: 16.043(4.547)). Complementary factors in the haemorrhagic group were: arterial hypertension (OR: -13.394(0.938)), mechanical ventilation (OR: 16.663(1.211)) and naso-gastric intubation (OR: 24.301(1.112)).

### Survival

**Figure 4:**
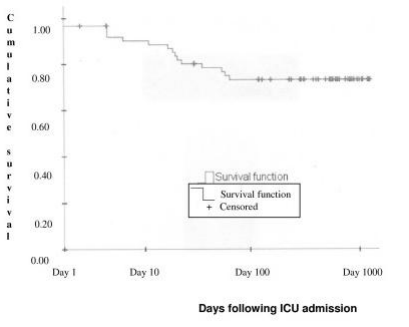
Kaplan Meier survival curve for severe stroke patients

Among the 62 patients studied, 16 (25.8%) died in the ICU and seven died in the year following hospital discharge. For the study period a Kaplan-Meier plot (Figure 4) showed a steady decline in patient numbers up to Day 100 with no further decline up to day 1000. The significant factors determined by univariate analysis influencing survival were: patient age, history of smoking and cardiac insufficiency, delay between onset of symptoms and ICU admission, APACHE II and GCS at hospital admission, hospital length of stay, thrombolysis, and the requirement for sedation, anti-epileptic treatment, osmotic diuresis, naso-gastric intubation, mechanical ventilation and surgery.

To evaluate the impact of "duration of ventilation" on prognosis, we divided this variable into 3 class categories; 0: no ventilation; 1: ventilation < 72 hours (short) and 2: ventilation > 72 hours (long). Univariate analysis of ventilation confirmed a bad prognosis with a risk ratio (RR) of 2.65 (95% CI: 1.55 to 4.55; p < 10-3). Multivariate analysis using a Cox regression provided the three final independent variables shown below (Table 4). Using a multivariate analysis we also examined the effect of each day of mechanical ventilation on worsening the CVA patients' prognosis (Table 4).

**Table 4:** Cox model for predicting survival

**Consideration**		**Risk Ratio**	**95% CI**	**p-value**
**Classes of mechanical ventilation**	GCS at hospital administration	0.79	0.66 to 0.95	0.012
	APACHE II at hospital admission	1.12	1.00 to 1.26	0.049
	Duration of mechanical ventilation (categorical)	2.45	1.36 to 4.43	0.003

**Number of days on mechanical ventilation**	GCS at hospital admission	0.80	0.66 to 097	0.024
	APACHE II at hospital admission	1.12	1.00 to 1.24	0.050
	Duration of mechanical ventilation (days)	1.07	1.01 to 1.15	0.046

## Discussion

Although the development of new stroke units and treatment modalities have reduced the disabilities and mortalities caused by acute severe strokes, the real clinical benefits for the most severely affected group of patients remain highly speculative. This study analyzed the benefits and implications of the therapeutic resources used in treating critical stroke patients (ischemic and haemorrhagic) in respect to their survival and functional abilities. Disability was measured by the ordinal variable SIS-16, but was better predicted by linear regression. Prognosis was studied by taking into consideration both the time factor and Kaplan-Meier survival tables.

For the study period, severe stroke accounted for 7% of medical ICU admissions and 17.5% of all hospital stroke cases. The decision to admit a patient into the ICU was determined primarily by their level of consciousness, and for the urgency to use intensive therapeutic procedures, such as mechanical ventilation, sedation, anti-epileptics, diuretics, osmotic or steroid treatment and surgical interventions. Pre-stroke the patients were completely independent with a Karnofsky score higher than 90 for both types of CVA. After examining the GCS on admission, we found an average score of less than 10 for both ischemic and haemorrhagic CVAs, which had probably influenced their ICU admission [24]. Once admitted, patients underwent intensive therapeutic procedures such as; thrombolysis, unblocking respiratory airways, mechanical ventilation, haemodynamic monitoring and treatment for intracranial hypertension.

Taking ICU admission as our starting point, important differences were noted between ischemic and haemorrhagic CVA. All risk factors, excluding atrial fibrillation, were identical in the two types of CVA [25,26]. We found that ischemic CVA was better tolerated as haemorrhagic CVA patients frequently required sedation in order to administer anti-epileptic treatment, surgical interventions, or osmotic diuretics. On admission the level of consciousness measured by the GCS was lower in ischemic CVA than in haemorrhagic CVA cases, and displayed a major increase between 48 hours post-CVA and ICU departure [27,28]. As previously seen in other studies [24,26,29-31] a strong improvement in functional ability (SIS-16/80) was noted after hospital departure and in the following 3 months, although this improvement was less pronounced at later times.

There are several factors that can predict the dependency status at hospital discharge, of these haemorrhagic CVA with a low level of consciousness on admission and a longer duration of hospital stay is associated with the worst functional ability. Moreover, on admission a severe haemorrhagic CVA with a GCS of 7 has more than 30 points less in the SIS-16 score at discharge than a severe ischemic CVA with a GCS of 9. Severe disability was identified in 40.5% of surviving patients on discharge, which decreased after one year to 24%. This result confirms the Barthel Index results of Navarrete-Navarro et al. [24], who showed that 26 % of severe stroke patients had a poor outcome and a severe functional disability after one year. Measuring neurological severity at hospital admission by GCS is the main determinant of future functional capacity in CVA patients at discharge and after one year. Indeed, although the patients' functional ability had improved after one year, it would be best predicted by their functional ability at discharge. Surviving severe stroke patients increase the burden of the disease, due to their health care utilization, which necessitates acute care, rehabilitation and an increased discharge rate to nursing homes, which all contribute to the increased cost of the disease [24,27,32,33].

Three independent factors contribute to the survival outcome of severe stroke patients. Of these, the decreased level of consciousness evaluated by GCS is the most important determinant of increased mortality. Mortality risk increases by 20% for each unit decrease in the GCS. Previous studies have confirmed the mortality prediction value of the GCS at admission, 30 days [28,34] and one-year later [24]. Moreover, a low GCS at admission coupled with an absence of pupillary light response corresponds to a poorer prognosis for survival [28].

Prognostic scoring systems such as APACHE can provide initial risk stratification for severely ill hospitalized patients. Studies have shown [17,24] that APACHE can predict survival outcome independently. Scoring the severity of the neurological disease and the severity of the patients general health status on admission are both good survival rate predictors in severe stroke patients [24].

Endotracheal intubation and the necessity to apply mechanical ventilation in severe stroke patients for neurological reasons are accompanied by the worst prognosis. Many studies have shown that stroke patients requiring mechanical ventilation have a bad outcome and surviving patients remain deeply disabled [28,34-37]. Santoli et al. showed that an assessment of brain stem reflexes might help identify the subgroup of patients with a high probability of death despite mechanical ventilation [36]. Moreover, a short duration of mechanical ventilation (< 72 hours) is still a bad predictor of survival with a risk ratio of 2.45 (95% CI: 1.36 to 4.43; p < 0.003). No difference between ischemic and haemorrhagic stroke patients was noted in terms of duration and requirement for mechanical ventilation. Indeed, when we looked at the ICU interventions our results revealed (Table 2) that all these factors were uninfluenced by the cause of the patients' CVA.

## Conclusion

When poorly selected patients are admitted into an ICU, inappropriate use of technology may not save lives, nor improve the quality of life, but rather transform dying into a prolonged, miserable and undignified process. We have shown that several factors and their assessments can help to identify the subgroup of patients that have an acutely reversible medical condition and are liable to have a potentially good functional recovery, from those that may suffer a prolonged and drawn out death regardless of any medical intervention. This raises important ethical questions: is it permissible to select certain patients for medical interventions and resources, and to condemn others to death without trying to intervene? Or, do these interventions constitute a therapeutic relentlessness that is an indispensable necessity?

The size of the cohort was limited as this study was conducted in only one centre over a 30 month period. Therefore, further studies involving a greater number of patients are urgently needed in order to shed some light on theses ethical and economical considerations.

## Abbreviations

ANOVA: Analysis of variance; APACHE: Acute Physiology, Age, Chronic Health Evaluation; CVA: Cerebrovascular accident; ICU: Intensive care unit; GCS: Glasgow Coma Scale; OR: Odds ratio; RR: Risk ratio; SD: Standard deviation; SIS: Stroke impact scale.

## Competing interests

We the authors declare that in the past five years have not received reimbursements, fees, funding, or salary from an organization that may in any way gain or lose financially from the publication of this manuscript, either now or in the future. There are no organizations financing this manuscript (including the article-processing charge). Do not hold any stocks or shares in an organization that may in any way gain or lose financially from the publication of this manuscript. Do not hold, or are currently applying for any patents relating to the content of this manuscript. We have you received reimbursements, fees, funding, or salary from an organization that holds or has applied for patents relating to the content of this manuscript. We do not have any other financial competing interests.

There are no non-financial competing interests (political, personal, religious, ideological, academic, intellectual, commercial or any other) to declare in relation to this manuscript.

## Authors' contributions

MR: wrote the protocol, applied it, collected the data and wrote the original document. SK: wrote the original document. SH–C: wrote the original document. GS: performed the statistics. FS: collected the data. GD: collected data. TB: collected data. ZA-B: collected data. GK: collected data.

## Pre-publication history

The pre-publication history for this paper can be accessed here:


